# Evaluating the Proportion of Foods and Beverages in the Canadian Grocery and Chain Restaurant Food Supply That Would Be Restricted from Marketing to Children on Television and Digital Media

**DOI:** 10.3390/nu17111828

**Published:** 2025-05-28

**Authors:** Hayun Jeong, Christine Mulligan, Ayesha Khan, Laura Vergeer, Mary R. L’Abbe

**Affiliations:** Department of Nutritional Sciences, Temerty Faculty of Medicine, University of Toronto, Toronto, ON M5S 1A8, Canada; hayun.jeong@mail.utoronto.ca (H.J.); christine.mulligan@mail.utoronto.ca (C.M.); ayeshaak.khan@mail.utoronto.ca (A.K.); laura.vergeer@utoronto.ca (L.V.)

**Keywords:** food advertising, food marketing, marketing restrictions, marketing to children, nutrient profiling

## Abstract

**Background/Objectives:** Despite evidence on the association between marketing unhealthy foods to children (M2K) and negative health outcomes, M2K remains widespread in Canada. To support mandatory restrictions, Health Canada has prioritized a monitoring strategy to assess the current state of M2K, identify gaps, and establish a baseline for future policy evaluation. This study aimed to support this initiative by updating the University of Toronto (UofT) Food Classification List and evaluating the proportion of foods and beverages in the Canadian grocery and restaurant food supply that would be permitted or restricted from M2K under Health Canada’s proposed nutrient profile model. **Methods:** Grocery items from the UofT Food Label Information Price 2020 (*n* = 24,949) and restaurant menu items from Menu-Food Label Information Price 2020 (*n* = 14,286) databases were evaluated using Health Canada’s M2K nutrient profile model, which assesses foods solely based on thresholds for added sodium, sugars, and saturated fat. The proportion of items permitted for or restricted from M2K was determined overall and by food and menu categories for grocery and restaurant items, respectively. **Results:** The updated UofT List contained *n* = 24,494 grocery items and *n* = 14,286 menu items. Overall, 83% (*n* = 32,664/39,235) of foods and beverages in the 2020 Canadian food supply would be restricted from M2K. Among grocery items, 23% (*n* = 5630) would be permitted and 77% (*n* = 19,202) would be restricted from M2K. Among restaurant items, only 6% (*n* = 837) would be permitted and 94% (*n* = 13,442) restricted. **Conclusions:** The updated UofT List supports Health Canada’s monitoring strategy and highlights the large proportion of unhealthy products in the Canadian food supply that are currently still permitted for M2K. While Health Canada’s M2K nutrient profile model is stringent, gaps remain that could allow continued M2K exposure under the current proposed policy. Ongoing monitoring and policy refinement are essential to effectively protect children from M2K and its harmful effects.

## 1. Introduction

As the Heart and Stroke Foundation highlights in their report [1], “The kids are not alright. How the food and beverage industry is marketing our children and youth to death”, children are exposed to an alarming amount of unhealthy food and beverage marketing, commonly referred to as marketing-to-kids (M2K), every day and everywhere [1]. M2K predominantly promotes junk-type foods high in sugars, sodium, and saturated fats [1,2], which contribute to dietary patterns associated with increased risks for diet-related diseases such as obesity, diabetes, and heart disease [3]. For example, over 50 million food and beverage ads appear on the top 10 most popular children’s websites each year, and over 90% of them are for ultra-processed foods, such as soft drinks, sweetened breakfast cereals, cookies, and chicken nuggets [4]. With this constant exposure to M2K that is strategically designed to appeal to children through the use of cartoons, characters, games, and other persuasive marketing techniques [5,6], brand loyalty builds among children and impacts the food they prefer and choose to purchase [5,7]. This then cascades into an increase in the consumption of unhealthy foods and a sustained energy imbalance, which ultimately leads to weight gain, increasing their risk for diet-related disease [8].

Recognizing these detrimental impacts, the World Health Organization (WHO) has recommended that all countries develop stringent policies that restrict M2K [5]. In Canada, Bill S-228, the Child Health Protection Act [9], was introduced in 2016, proposing to federally mandate the restriction of M2K to children under the age of 13 in a variety of media and settings. However, the Bill failed to pass in June 2019, and the associated draft regulations from Health Canada were not implemented. More recently, a second attempt to enact legislation regulating M2K, Bill C-252 (an Act to amend the Food and Drugs Act: prohibition of food and beverage marketing directed at children), was introduced into the House of Commons and is currently at third reading in the Senate [10]. The new Bill proposes to restrict the advertising to children of foods with excessive levels of sugars, sodium, or saturated fats for children (i.e., exceed thresholds established by Health Canada), with the intent to help reduce Canadian children’s overconsumption of these nutrients-of-concern, as they are associated with an increased risk of developing diet-related chronic disease [11].

The WHO also states that policy frameworks for restricting M2K should include systems for monitoring and evaluation to ensure the overall aims and objectives are being met [5]. Accordingly, as Canada awaits the passing of Bill C-252, Health Canada has prioritized the development and implementation of their M2K Monitoring Strategy in order to track the evolving food marketing landscape, collect baseline data, and monitor the immediate (e.g., the frequency and power of M2K) and long-term effects (e.g., changes in children’s awareness to food marketing, food intake, and reduction in ill health effects) of the policy once implemented [8,12]. Currently, Health Canada is monitoring the media and settings where children spend the most time (e.g., schools, recreation facilities) and are most likely to be exposed to food advertising (e.g., television, digital media, packaging, restaurants, and retail settings). In addition, marketing techniques that are most prominently used in M2K are being closely monitored. Through their monitoring, Health Canada aims to strengthen our understanding of the current state of food advertising to children in Canada, identify gaps, and construct a baseline of Canadian data that can be used to measure changes over time, which, together, can support evidence-informed policy development [8].

As part of the ongoing development of Health Canada’s M2K Monitoring Strategy, protocols to improve consistency across monitoring activities were needed, prompting the development of the Protocol for classifying foods using Health Canada’s nutrient profile model for advertising restrictions (hereafter referred to as “M2K NPM”) released in 2021 [8,13]. The goal of this Protocol was to provide methodological guidance and support, and improve consistency in monitoring research efforts across Canada, particularly in classifying foods based on their nutritional quality. The original Protocol was largely based on the University of Toronto (UofT) Food Classification List, which identified foods and beverages in the 2013 Canadian food supply that would be either permitted for or restricted from M2K under Health Canada’s M2K NPM [13,14]. However, this list is outdated, especially considering it reflects the pre-pandemic food supply. Thus, an updated UofT Food Classification List to better represent the current Canadian food supply is warranted.

Therefore, the objectives of this study were to (1) update the UofT Food Classification List to support Health Canada’s Protocol for classifying foods using the M2K NPM, and (2) determine the proportion of foods and beverages in the Canadian grocery and restaurant food supply that would be permitted for or restricted from M2K.

## 2. Materials and Methods

### 2.1. Study Design

A cross-sectional analysis of the Canadian grocery and chain restaurant food supply was conducted using the UofT’s Food Label Information and Price (FLIP) 2020 [15] and Menu-FLIP 2020 [16] databases, respectively. Items were evaluated using Health Canada’s M2K NPM [8,13].

### 2.2. University of Toronto Food Classification List

The UofT Food Classification List was first developed as part of Health Canada’s Protocol, released in 2021. The List was originally based on the FLIP 2017 and Menu-FLIP 2016 databases [14], which contain nutritional composition information for more than 17,000 packaged foods from major grocery retailers and more than 12,000 menu items gathered from national chain restaurants, respectively. Foods in the FLIP databases were assessed according to Health Canada’s M2K NPM to identify products that would be permitted for or restricted from M2K under the current proposed regulations.

The List is the first key component of Health Canada’s Protocol as stakeholders can easily search the List for the specific food, main dish or individual meal/multi-food component(s) featured in an advertisement, and, if listed, can determine if the item would be permitted for or restricted from M2K [13]. However, since the Protocol release, more recent versions of the FLIP databases have become available (i.e., FLIP 2020 and Menu-FLIP 2020), and, accordingly, this study applied Health Canada’s M2K NPM to the 2020 databases to update the UofT Food Classification List.

### 2.3. Food Label Information and Price (FLIP) 2020 Database

FLIP 2020 is a branded food composition database that contains web-scraped (an automated process used for extracting data from websites implemented using a bot or a web crawler [15]) product information for over 70,000 grocery food and beverage items (hereinafter referred to as “grocery food items”) sold in seven major grocery retailers (*n* = 48,829 unique products), representing more than 80% of the Canadian grocery market share [15]. Unlike previous collections, FLIP 2020 contains fresh (i.e., fresh fruit and vegetables, and unprocessed meats) and packaged products. A detailed description of FLIP 2020 is explained elsewhere [15]. Briefly, the FLIP 2020 database was collected between May 2020 and February 2021 and contains information on the product name, UPC, brand, nutritional composition as shown on the Nutrition Facts table (NFt), ingredients list, container size, price (regular and sale price), and images of the product packaging (as available). All products in FLIP are categorized according to the major (*n* = 24) and minor (*n* = 153) food categories in Health Canada’s Table of Reference Amounts for Foods (TRA), which are used in Canada’s nutrition labelling regulations to determine the standard serving size based on amounts typically consumed by Canadians in a single sitting [17].

### 2.4. Menu-Food Label Information and Price (Menu-FLIP) 2020

Menu-FLIP 2020 is a database containing the nutritional composition information of Canadian chain restaurant menu items. Details of the database are provided elsewhere [16], but briefly, the database includes 18,760 menu items from 141 chain restaurants, representing over 70% of the 2020 Canadian food service market share. Nutrition information was extracted from online menus by either an optical character recognition tool or manually by research assistants in November and December of 2020. Items in Menu-FLIP were categorized as one of the following major menu categories: beverages, desserts, entrées, sides, and starters, and further broken down into subcategories.

### 2.5. Health Canada’s Proposed Nutrient Profile Model for Advertising Restrictions (M2K NPM)

**Figure 1:**
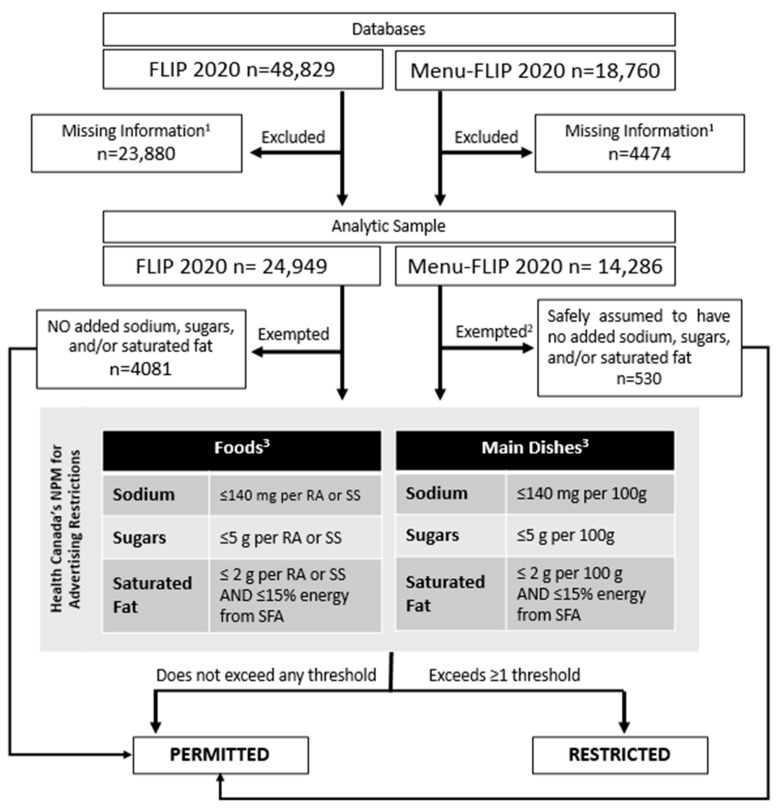
Flowchart for identifying foods and beverages in FLIP and Menu-FLIP 2020 that would be permitted for or restricted from M2K based on Health Canada’s Proposed Nutrient Profile Model for Advertising Restrictions. ^1^ Items classified as Foods, not exempted, and missing information for all three nutrients were excluded. Items classified as Main Dishes, not exempted, and missing information for all three nutrients or serving size were excluded. ^2^ As ingredient lists were unavailable in Menu-FLIP 2020, only items that were reasonably assumed to have no added nutrients-of-concern were exempted. ^3^ Adapted from Health Canada’s Monitoring Protocol [13], where Main Dishes are items with reference amounts above 200 g. All other items are classified as Foods. Abbreviations: FLIP—Food Label Information and Price database; NPM—nutrient profile model; SS—serving size; RA—reference amount; SFA—saturated fatty acids; M2K—unhealthy foods/beverage Marketing-to-Kids.

Applying Health Canada’s M2K NPM is described extensively elsewhere [13,14] and summarized in Figure 1. Briefly, foods and beverages containing added sodium, free sugars, or fat are evaluated against the respective nutrient thresholds, with each nutrient evaluated independently. These thresholds correspond to 6% of the Daily Value (DV) for sodium, 5% for sugars, and 10% for saturated fat, based on the reference amount specified for each Health Canada’s TRA item. Foods and beverages exceeding one or more of these thresholds are restricted from M2K, whereas those that do not exceed any thresholds are permitted for M2K. This classification is based exclusively on thresholds for added sodium, free sugars, or fat, and does not account for nutrient density (e.g., fibre, protein, or micronutrient content). Standalone sodium, sugars, and fat products like table salt, granulated sugar, and unsalted butter, are exempt from evaluation for their respective nutrient thresholds, while foods containing no added sodium, free sugars, or added fat (e.g., whole or cut vegetables/fruit, whole grains, plain milk, plain yogurt, eggs) are completely exempt from evaluation.

For menu items in Menu-FLIP, where ingredient lists are unavailable, only items reasonably assumed to contain no added sodium, sugars, or fat are exempted (e.g., raw apple slices, bottled water). Items with small reference amounts of 30 g or less (e.g., sauces, dips or condiments) were assessed based on 50 g amounts [13]. Items with standard reference amounts exceeding 30 g were evaluated against either the reference amount or the serving size stated on the NFt or menu, whichever was greater. Items classified as main dishes (e.g., restaurant meals, such as hamburgers, chicken tenders, pizza) with a reference amount of 200 g or greater were assessed based on 100 g amounts. When restaurant meals contained multiple components (e.g., entrée, side), each component was assessed against the nutrient thresholds individually.

### 2.6. Analytic Sample

Some items in FLIP 2020 were excluded from the UofT Food Classification List and analysis because of missing or implausible information required to apply Health Canada’s M2K NPM. Items without a TRA reference amount were excluded (*n* = 926). Items with missing nutrition information or implausible nutrient information, as determined by Atwater calculations >20% from declared caloric values, were excluded (*n* = 22,837). An additional 117 items were excluded as they were missing information for all three nutrients-of-concern required to apply the M2K NPM. The final UofT Food Classification List and analytic sample for grocery foods and beverages included 24,949 items.

Some menu items in Menu-FLIP 2020 that were not exempted but were missing information required to apply Health Canada’s M2K NPM were excluded. Items classified as “entrées” and missing information for all three nutrients or serving sizes were excluded (*n* = 3886). Items classified as all other major menu categories and missing information for all three nutrients were excluded (*n* = 588). The final UofT Food Classification List and analytic sample for restaurant foods included 14,286 items.

### 2.7. Statistical Analysis

Items exceeding one or more of Health Canada’s proposed thresholds for restrictions on M2K for sodium, saturated fat, and/or sugars were considered restricted from M2K. Exempt items and those that were evaluated not to exceed any of the thresholds were considered permitted for marketing. The number and proportion of items in the updated UofT Food Classification List that would be exempted from, permitted for, or restricted from M2K restrictions were calculated overall and by TRA categories for grocery items and major menu categories for menu items. The number and proportion of items exceeding the individual thresholds for each nutrient were also determined.

## 3. Results

### 3.1. Updated UofT Food Classification List

The updated UofT Food Classification List identifies foods and beverages in the 2020 Canadian food supply that would be exempted from, permitted for, or restricted from M2K restrictions. It includes 24,949 grocery items from FLIP 2020 and 14,286 menu items from Menu-FLIP 2020. The List is stored within Health Canada’s Monitoring food marketing to children: a protocol for classifying foods, Version 1.1. 2023 [13]. It can be found through Health Canada and the Public Health Agency of Canada’s collaborative SharePoint site. Currently, the List, as part of the Protocol, is only accessible to Health Canada staff and research collaborators, as its primary purpose is to facilitate consistent data collection and interpretation that is comparable across Health Canada’s various monitoring activities and over time, and not for compliance monitoring or enforcement activities.

### 3.2. Applying Health Canada’s M2K NPM to the 2020 Canadian Food Supply

Overall, 83% (*n* = 32,644/39,235) of foods and beverages in the Canadian grocery and chain restaurant food supply would be restricted from M2K (Table 1). A total of 60% (*n* = 23,562) of items exceeded the sodium threshold, 38% (*n* = 14,896) exceeded the saturated fat threshold, and 42% (*n* = 16,633) exceeded the sugars threshold. On the other hand, 12% (*n* = 4611) would be exempt from all thresholds (i.e., did not contain any added sodium, fat, or sugars) and automatically be permitted for M2K, and 5% (*n* = 1856) did not exceed any of the thresholds, for a total 17% (*n* = 6467) that would be permitted for M2K (Table 1).

### 3.3. Canadian Grocery Supply: FLIP 2020

A total of 16% (*n* = 4081) of grocery food items would be exempt from Health Canada’s proposed M2K restrictions, and 6% (*n* = 1549) of products did not exceed any of the thresholds. Altogether, 23% (*n* = 5630) of grocery items would be permitted for M2K (Table 1). A total of 77% (*n* = 19,202) of grocery food items would be restricted from M2K, with 51% (*n* = 12,599) of products exceeding the sodium threshold, 22% (*n* = 5456) exceeding the saturated fat threshold, and 42% (*n* = 10,577) exceeding the sugars threshold (Table S1).

**Figure 2:**
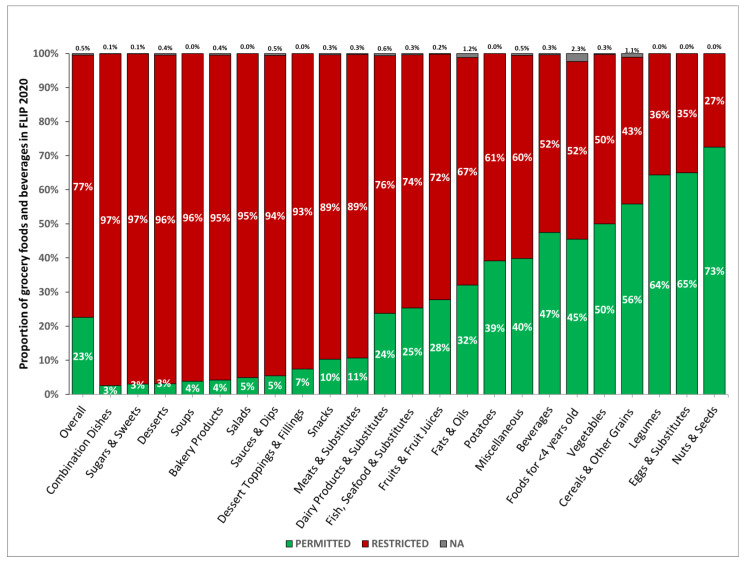
Proportion of grocery food items in the 2020 Canadian food supply that would be permitted for or restricted from M2K by TRA category. *n* = 24,949. Food and beverage items in FLIP 2020 were categorized into food categories as defined in Health Canada’s Table of Reference Amounts for Foods (TRA) [17]. Products with insufficient nutrition information to determine if they would be permitted for or restricted from M2K were classified as NA. For example, if a product did not meet the exemption criteria, had nutrition information for serving size, sodium, and sugars available and did not exceed thresholds for either nutrient, but was missing information for saturated fat, the product could not be positively categorized as permitted for or restricted from M2K; thus, was classified as NA. Totals may not equal 100% because of rounding. Abbreviations: FLIP—Food Label Information and Price database; M2K—unhealthy foods/beverage Marketing-to-Kids; TRA—Table of Reference Amounts for Foods.

Very few food categories (5/22 categories) had greater than 50% of products that would be permitted for M2K (Figure 2): vegetables (50%), cereals and other grains (56%), legumes (64%), eggs and substitutes (65%), and nuts and seeds (73%). On the other hand, almost half of all food categories had over 89% of products that would be restricted from M2K: meats and substitutes (89%), snacks (89%), dessert toppings and fillings (93%), sauces and dips (94%), salads (95%), bakery products (95%), soups (96%), desserts (96%), sugars and sweets (97%), and combination dishes (97%).

Among food categories with greater than 50% of products permitted for M2K, the sodium threshold was exceeded the most compared to saturated fat and sugar thresholds. Otherwise, the categories with the highest proportion of grocery items that would exceed thresholds varied by nutrient. For meats and substitutes, snacks, sauces and dips, salads, soups, combination dishes, and bakery products, sodium was the most common threshold exceeded, with over 70% of items in these categories exceeding the sodium threshold. For dessert toppings and fillings, sugars and sweets, and desserts, the sugar threshold was exceeded the most, with over 92% of items in these categories exceeding the sugar threshold.

### 3.4. Canadian Chain Restaurant Food Supply: Menu-FLIP 2020

A total of 4% (*n* = 530) of chain restaurant menu items were reasonably assumed to contain no added sodium, sugars, or fat, and were exempt from all of Health Canada’s proposed thresholds for restrictions on M2K. A total of 2% (*n* = 307) of menu items did not exceed any of the thresholds. Overall, 6% (*n* = 837) of menu items would be permitted for M2K (Table 1). A total of 94% (*n* = 13,442) of menu items would be restricted from M2K, with 82% (*n* = 10,963) exceeding the sodium threshold, 70% (*n* = 9440) exceeding the saturated fat threshold, and 45% (*n* = 6056) exceeding the sugars threshold (Table 1).

**Figure 3:**
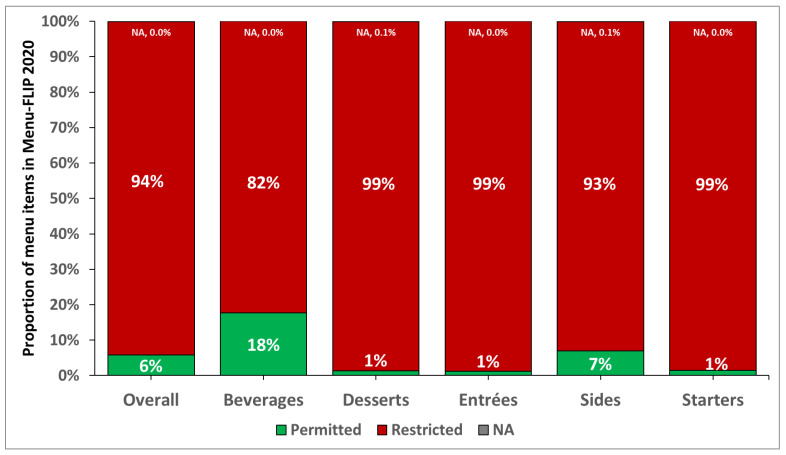
Proportion of chain restaurant menu items in the 2020 Canadian food supply that would be permitted for or restricted from M2K by major menu category. *n* = 14,286. Chain restaurant menu items in Menu-FLIP 2020 were categorized into major menu categories [16]. Menu items with insufficient nutrition information to determine if they would be permitted for or restricted from M2K were classified as NA. For example, if a menu item could not be reasonably assumed to have no added nutrients-of-concern, had nutrition information for serving size, sodium, and sugars available and did not exceed thresholds for either nutrient, but was missing information for saturated fat, the menu item could not be positively categorized as permitted for or restricted from M2K; thus, was classified as NA. Totals may not equal 100% because of rounding. Abbreviations: Menu-FLIP—Food Label Information and Price chain restaurant menu database; M2K—unhealthy foods/beverage Marketing-to-Kids.

Only two menu categories contained greater than 5% of menu items permitted for M2K (Figure 3): sides (7%) and beverages (18%), whereas 99% of menu items in desserts, entrees and starters would be restricted from M2K.

Unsurprisingly, the categories with the highest proportion of menu items that would exceed thresholds varied by nutrient (Table S2). Menu items in the entrées, sides, and starters categories exceeded sodium thresholds most often, with 97%, 96%, and 99.8% of menu items in these categories exceeding the sodium threshold, respectively. For menu items in the beverages and desserts categories, the sugar threshold was exceeded the most, with 99% and 95% of menu items in these categories exceeding the sugar threshold, respectively.

## 4. Discussion

The Canadian food supply, including both grocery food and beverage items and chain restaurant menu items, would be largely restricted from M2K on television and digital media under Health Canada’s proposed M2K NPM. A total of 24,949 grocery food items and 14,286 menu items were included in the updated UofT Food Classification List to better reflect the current Canadian food supply and to inform the continued monitoring of M2K across a range of media and settings in Canada. Overall, 77% of grocery food items and 94% of chain restaurant menu items exceeded at least one of Health Canada’s M2K NPM nutrient thresholds, and would therefore be restricted from M2K. These findings align with other research indicating high levels of nutrients-of-concern in the foods available in the Canadian food supply that are nevertheless being heavily marketed to children [6,14].

This study was conducted to inform the development of Health Canada’s M2K Monitoring Strategy by providing an updated UofT Food Classification List as part of their protocol for classifying foods based on Health Canada’s M2K NPM. According to the Food and Beverage Marketing Monitoring Framework for Canada [12], data on three main outcome measurement indicators are needed to monitor the impacts of M2K restrictions: (1) food marketing indicators, (2) company-level indicators, and (3) behavioural- and health-related indicators. The updated UofT List, which leverages branded food composition databases, is an integral tool for monitoring the first two indicators. Food marketing indicators include children’s actual and potential exposure to the frequency of M2K in various media and settings [12,18]; the power (i.e., the techniques being used) of M2K; and the healthfulness of the products that are being marketed. The updated UofT List can also aid in monitoring some company-level indicators, such as companies’ compliance with the M2K restrictions, changes in the types of food and beverage items sold, and any company-driven reformulation. Furthermore, continued updating of the UofT List is needed to compare outcomes pre-policy implementation (current List) and at varying intervals post-implementation to capture both the short and long-term impacts of M2K restrictions. The UofT List contributes to monitoring M2K and Health Canada’s overall role both in developing evidence-informed nutrition policy and studying its impact post-implementation.

It is also important to emphasize that the UofT List plays a crucial role in standardizing M2K monitoring across Canada. Given that the frequency, nature, and impact of M2K and children’s exposure to marketing vary significantly by region and media [12,19], it is essential to collect food data that are representative of all regions across Canada. As the FLIP databases that underpin the UofT list include foods and beverages from the leading food retailers nationwide, the UofT List ensures that data collection and analysis are consistent, enabling reliable comparisons and interpretations of findings across different regions and media in Canada. Furthermore, the FLIP databases are regularly enhanced and updated to capture changes in the food supply [15], exemplified by the addition of fresh fruit and vegetables as well as fresh meats in the current collection, which can ensure that UofT List also remains relevant and accurately identifies foods and beverages that would be permitted for or restricted from M2K.

Our results indicate that Health Canada’s M2K NPM would likely be effective in identifying foods and beverages that are generally considered unhealthy and should not be promoted to children. Only 5 packaged food categories had more than 50% of products permitted for M2K: vegetables, cereals and other grains, legumes, eggs and substitutes, and nuts and seeds. These categories are typically categorized as “healthy” by existing nutrient profiling models [20], such as Food Standards Australia New Zealand’s (FSANZ) nutrient profiling scoring criterion and France’s Nutri-score system, and align with the 2019 Canada’s food guide [21] among other dietary guidelines. Furthermore, the proposed restrictions effectively differentiate between “nutritious foods” and “foods that undermine healthy eating” [11,21] within these categories based on criteria consistent with the descriptions of nutritious foods in Canada’s Dietary Guidelines (i.e., have little to no added sodium and saturated fat, and little to no free sugars; should not contribute to excess consumption of sodium, free sugars, or saturated fat [11]). The proposed M2K restrictions also align with Canada’s Healthy Eating Strategy initiatives, which aim to improve the overall health of Canadians by creating supportive food environments that make the healthier choice the easier choice. In particular, M2K restrictions align with one of the Healthy Eating Strategy’s primary goals of protecting vulnerable populations [22]. Children are particularly vulnerable to food advertising [5,23], as it can easily influence their food attitudes, preferences, purchase requests, dietary patterns, and, ultimately, their overall health, and they are exposed to a high amount of M2K. The Healthy Eating Strategy, therefore, commits to protecting the health of this vulnerable population by restricting M2K.

Conversely, at least 89% of products in other categories, especially those that have been identified to constitute a large proportion of products currently being marketed to children [6,14], such as snacks, bakery products, desserts, dessert toppings and fillings, and sugars and sweets, would be restricted from M2K. Given the broad reach of M2K on digital media and other settings, such as in retail stores, product packaging, and recreation facilities for unhealthy food products [4], our findings support the need to expedite the implementation of these restrictions. Furthermore, expanding the current proposed regulations that only apply to digital media to broader settings, as listed above, can further help children make healthier food choices.

A previous analysis of the 2013 FLIP database found that 82% of packaged foods in the grocery food supply were not exempt from evaluation and exceeded at least one threshold in Health Canada’s M2K NPM [14] and therefore would be restricted from marketing to children. In comparison, our updated analysis found that 77% of items in the 2020 grocery food supply would be restricted. While this decrease may suggest a modest improvement in the nutritional quality of the food supply, it is more likely attributable to the expanded scope of the FLIP database to include fresh fruits and vegetables—items that are typically exempt from evaluation and permitted under the M2K NPM. As such, the still high proportion of grocery food items exceeding nutrient thresholds also highlights the continued need for strong, mandatory restrictions on M2K to address the persistent presence of unhealthy foods in the Canadian food environment.

For the chain restaurant sector, 94% of menu items exceeded at least one nutrient threshold, and would therefore be restricted from M2K under proposed regulations. These results agree with literature suggesting that most Canadian menu items contain excessive levels of nutrients-of-concern [16,24,25], with little to no progress in improving their nutritional quality [24]. This poor nutritional quality of chain restaurant menu items appears to be a global problem, with over 95% of menu items in the US and UK also containing high levels of at least one nutrient-of-concern [26]. This is a major concern, as surveys suggest that approximately seven in ten school-aged children in Canada eat at fast-food restaurants once a month, and about one in four do so once a week [27], with parents largely driven by children’s preferences when eating out together [8,28]. In addition, studies have found that there are numerous child-appealing advertisements with cartoon or TV/movie characters for most fast-food menu items, with very few healthy entrée options, in contrast to the large number of sugar-sweetened beverage and dessert options on the children’s menu in fast-food restaurants [4,29,30]. The vast majority of these children’s meals in restaurants do not meet nutrition standards [29,30,31]. Our results also show that 95% of menu items categorized as children’s meals by the establishments exceed at least one proposed nutrient threshold for restrictions on M2K. Nevertheless, marketing these menu items to children remains pervasive in Canada through various media platforms and at point-of-sale in restaurants [4,8]. The Heart and Stroke Foundation of Canada reported that 41% of ads outside over 2000 restaurants used child-appealing marketing [4], with restaurants accounting for the highest marketing expenditures in the food industry [31]. Historically, restaurant foods have been largely overlooked by other Canadian food policies, which have primarily focused on packaged foods and beverages sold in retail environments [22,25,32]. This oversight has allowed the restaurant sector to evade the same level of scrutiny and regulatory restrictions faced by grocery retail environments, despite its significant influence on children’s diets. Given the substantial role that restaurant foods play in the diets of Canadian children, we must ensure that this sector is not continuously overlooked when it comes to protecting our most vulnerable populations. These findings underscore the urgent need for regulating M2K in the restaurant sector to protect children and incentivize establishments to improve the nutritional quality of menu items, as well as the need for food policies in Canada to target restaurants in addition to grocery retail environments.

When examining nutrient thresholds individually, sodium was the most frequently exceeded nutrient for both grocery food and chain restaurant menu items in the Canadian food supply. This finding is significant given that sodium intake among Canadian children is substantially higher than recommended levels [33]. The National Academies of Sciences, Engineering, and Medicine (NASEM) recommend a maximum of 1200 mg/day for children aged 9–13, and 1500 mg/day for children aged 14 and older [34], while the WHO recommends the maximum sodium intake level of 2000 mg/day in adults be adjusted downward, based on the estimated energy requirements of children compared with adults [35]. However, the most recent national nutrition survey in Canada, the 2015 Canadian Community Health Survey (CCHS), revealed that 49% of children aged 1 to 3 years and 72% of children aged 4 to 8 years were consuming sodium above these thresholds [36], with older children and adolescents showing even higher intakes. High sodium consumption is linked to increased blood pressure [37], and recent data suggest that 7% of Canadian children have either borderline or overt hypertension [36]. Further, hypertension in children is associated with other risk factors for cardiovascular disease, including hyperlipidemia and insulin resistance, and children can also experience target organ damage from hypertension [36,38]. Reducing dietary sodium has been shown to lower blood pressure in children [39], and thus, the proposed regulations that permit only foods with sodium levels that are ≤6%DV for M2K may serve as an effective policy tool for reducing sodium intake among Canadian children.

While the proposed nutrient criteria for restrictions on M2K are promising in their ability to limit M2K to products with low levels of nutrients-of-concern, there are notable limitations to the regulatory framework that may impact its overall effectiveness. The proposed regulations, as described in the most recent policy update (June 2023) [8] and forward regulatory plan (December 2023) [40], intend to limit children’s exposure to M2K in television and digital media where children spend much of their time and are highly exposed to food advertising. While the policy update acknowledges that children are exposed to M2K in other types of media and settings, as well as techniques, the scope of the current proposed regulations fails to address product packaging and point-of-sale marketing, such as child-appealing posters/signs in restaurants and toy premiums included with children’s meals, despite these techniques being prominent if not top sources of children’s exposure to food marketing [6,41]. This critical gap may be a result of industry lobbying, where industry stakeholders are typically at an advantage in terms of influencing nutrition policy because of many strong, direct relationships and contact with decision-makers [42,43]. An analysis of Health Canada’s “Meeting and correspondence on healthy eating” database that details interactions between stakeholders and Health Canada related to nutrition policies revealed that >84% of meetings were held by industry stakeholders [42]. These industry stakeholders had five times more communications with Health Canada than non-industry stakeholders during the consideration of the first proposed bill to restrict M2K (Bill S-228), which eventually died in parliament. Similar lobbying efforts may be presumed for the current Bill C-252, given that 60% of industry-affiliated stakeholders explicitly registered to lobby on the topic of M2K restrictions between September 2016 and January 2021 [43].

Voluntary actions on M2K by the industry have consistently proven inadequate in curbing the promotion of unhealthy foods to children [44]. Despite their pledges to self-regulate, these efforts often lack transparency, enforceability, and coverage, resulting in minimal impact on reducing children’s exposure to foods high in nutrients-of-concern [44]. For example, in the United States, the Children’s Food and Beverage Advertising Initiative, a self-regulatory program, has been criticized for its limited scope and lenient nutritional criteria, allowing many unhealthy products to continue being marketed to children [45]. Similarly, in Australia, voluntary codes have been shown to be ineffective, with research indicating that children are still widely exposed to unhealthy M2K despite industry promises to reduce such marketing [46]. In contrast, countries that have implemented mandatory regulations have seen more promising outcomes. For instance, Chile’s strict advertising restrictions that prohibit the marketing of foods high in sugar, sodium, and saturated fat to children, and include specific restricted hours for this type of advertising across all TV channels in their regulations, have led to significant reductions in children’s exposure to unhealthy food marketing [47]. The success of such mandatory policies, coupled with the consistent failure of voluntary industry actions to effectively curb M2K, underscores the urgent need for Canada to adopt similar mandatory regulations. Without enforceable and broadly applicable regulations, the food industry is unlikely to make meaningful changes, and children will continue to be at risk of exposure to M2K that promotes poor dietary habits. This will be particularly important to support policy coherence with Canada’s forthcoming front-of-package labelling (FOPL) regulations in Canada that require all products high in sodium, sugars, or saturated fat to display a “high in” nutrition symbol beginning in January 2026 [48]. While mandatory FOPL has demonstrated positive impacts on consumer understanding and food choices [49], including among individuals with lower literacy, like children, if M2K prevails on product packaging, it could undermine the intended effect of the “high in” nutrition symbol by encouraging selection of products that should otherwise be avoided. Therefore, in order to ensure the effectiveness of M2K restrictions in reducing Canadian children’s exposure to foods high in nutrients-of-concern, the final regulations need to be mandatory, requiring all foods to meet the proposed nutrient criteria before being permitted for M2K, and should regulate all major sources of children’s exposure to food marketing, including product packaging.

This study presents an updated and more comprehensive evaluation of the Canadian food supply in relation to Health Canada’s proposed restrictions on M2K using recent FLIP databases, FLIP 2020 and Menu-FLIP 2020. The FLIP 2020 database also provides a more complete reflection of the grocery food supply, as it includes packaged items, fresh fruit and vegetables, and unprocessed meats for sale across seven major grocery retailers in 2020, which were not included in previous iterations. However, there are some inherent limitations to our study. FLIP is a cross-sectional database representing the Canadian food supply at the time of collection. As our data was collected in 2020, it is possible that there have been some changes in the food supply since then, especially due to various events such as the COVID-19 pandemic and the introduction of tariffs. Newly introduced grocery food or menu items and reformulated items would not have been captured, and those that have been entirely removed from the food supply would still have been included in this analysis. However, reformulation in the packaged food supply is uncommon, including in Canada, with previous studies showing that only a small proportion of packaged foods and beverages demonstrated changes in sodium or sugar content over time [50]. Additionally, FLIP 2020 and Menu-FLIP 2020 data are not sales-weighted, and analyses of Canadians’ purchasing behaviours of grocery food and menu items were not within the scope of this research. Lastly, due to the lack of regulations requiring nutrition labelling for online grocery retail and the restaurant sector, nutrition information is missing on some grocery retailers and chain restaurant websites, and even if present, the information is not standardized, which results in limited availability.

## 5. Conclusions

The UofT Food Classification List, updated through this research, informs the classification of foods in the Canadian food supply based on Health Canada’s M2K NPM and supports monitoring efforts in various media and settings nationwide. The updated UofT List also contributes to the baseline pre-policy data, which can be compared against post-policy data to assess the impacts of M2K restrictions. Furthermore, this study builds on the growing body of literature highlighting the high proportion of unhealthy foods that are being marketed to children. Our findings suggest that while Health Canada’s M2K NPM is strict, the application of the proposed policy leaves room for many loopholes for the continued exposure of M2K and its harmful effects on children. Therefore, to maximize the effectiveness of the policy, it is essential to address the gaps related to marketing in other media types, product packaging, and point-of-sale promotions. The implementation of the restrictions with some refinement and a comprehensive monitoring plan could further reduce children’s exposure to unhealthy foods and support healthier dietary habits. Continued research and monitoring are vital to evaluate the impact of these regulations and adapt strategies as necessary to protect our children.

## Figures and Tables

**Table 1:** Summary of the overall number and proportion of grocery store food and menu items in the 2020 Canadian food supply that would be permitted for or restricted from M2K.

			Permitted ^2^			Restricted ^2^			
Database	Total *n*	NA ^1^, *n* (%) ^2^	*n* (%)	Exempt, *n* (%)	Below M2K NPM ^3^, *n* (%)	*n* (%)	Exceed SOD, *n* (%) ^4^	Exceed SATFAT, *n* (%) ^4^	Exceed SUG, *n* (%) ^4^
FLIP 2020	24,949	117 (0.5%)	5630 (22.6%)	4081 (16.4%)	1549 (6.2%)	19,202 (77%)	12,599 (50.5%)	5456 (21.9%)	10,577 (42.4%)
Menu-FLIP 2020	14,286	7 (0.05%)	837 (5.9%)	530 (3.7%)	307 (2.1%)	13,442 (94.1%)	10,963 (81.6%)	9440 (70.2%)	6056 (45.1%)
Overall	39,235	124 (0.3%)	6467 (16.5%)	4611 (11.8%)	1856 (4.7%)	32,644 (83.2%)	23,562 (60.1%)	14,896 (38%)	16,633 (42.4%)

^1^ Foods and beverages with insufficient nutrition information to determine if they would be permitted for or restricted from M2K were classified as NA. For example, if a product did not meet the exemption criteria, had nutrition information for serving size, sodium, and sugars available and did not exceed thresholds for either nutrient, but was missing information for saturated fat, the product could not be positively categorized as permitted for or restricted from M2K; thus, was classified as NA. ^2^ Percentage of total foods and beverages analyzed in that database. ^3^ Items that were below all three nutrient thresholds listed in Health Canada’s M2K NPM [13,14] were classified as permitted for M2K. ^4^ For the three nutrients, totals exceed 100% as some items exceed M2K NPM for more than one nutrient. Abbreviations: FLIP—Food Label Information and Price database; M2K—unhealthy foods/beverage Marketing-to-Kids; M2K NPM—Health Canada’s proposed nutrient profile model for advertising restrictions; SOD—sodium threshold; SATFAT—saturated fat threshold; SUG—sugars threshold.

## Data Availability

The data from this study is not publicly available as they were sourced from the University of Toronto’s Food Label Information and Price Program. Interested researchers can request access to the datasets after submitting a request. Detailed access information is available on the FLIP Program website (https://labbelab.utoronto.ca/projects/food-label-information-price-flip/ accessed on 23 May 2025).

## Supplementary Materials

The following supporting information can be downloaded at: https://www.mdpi.com/article/10.3390/nu17111828/s1, Table S1. Summary of the proportion of grocery food items in the 2020 Canadian food supply that would exceed each nutrient threshold listed in Health Canada’s M2K NPM [8] across TRA categories; Table S2. Summary of the proportion of chain restaurant menu items in the 2020 Canadian food supply that would exceed each nutrient threshold listed in Health Canada’s M2K NPM [8] across menu categories.

## Abbreviations

The following abbreviations are used in this manuscript:

M2K	Marketing-to-kids
WHO	World Health Organization
M2K NPM	Health Canada’s nutrient profile model for advertising restrictions
UofT	University of Toronto
FLIP	Food Label Information and Price database
NFt	Nutrition Facts table
TRA	Table of Reference Amounts for Foods
DV	Daily Value
FSANZ	Food Standards Australia New Zealand
NASEM	National Academies of Sciences, Engineering, and Medicine
CCHS	Canadian Community Healthy Survey
