# Supplementary material for: Evaluating the Proportion of Foods and Beverages in the Canadian Grocery and Chain Restaurant Food Supply That Would Be Restricted from Marketing to Children on Television and Digital Media
Source: Nutrients. 2025 May 28;17(11):1828. doi: 10.3390/nu17111828 (PMC12156963; doi:10.3390/nu17111828)
Supplement: Supplementary file 1 [file nutrients-17-01828-s001.zip › nutrients-3649924-supplementary.pdf]

Supplementary Table S1. Summary of the proportion of grocery food items in the 2020 Canadian food supply that would exceed each nutrient threshold listed in Health Canada's M2K NPM [8] across TRA categories.

			Permitted[3]			Restricted[3]			
**TRA Category[1]**	**Total n**	**NA[2], n[3] (%)**	**n (%)**	**Exempt, n (%)**	**Below M2K NPM[4], n (%)**	**n (%)**	**Exceed SOD, n (%)[5]**	**Exceed SATFAT, n (%)[5]**	**Exceed SUG, n (%)[5]**
Bakery Products	3633	16 (0.4%)	153 (4.2%)	47 (1.3%)	106 (2.9%)	3464 (95.3%)	2517 (69.3%)	1387 (38.2%)	2173 (59.8%)
Beverages	1482	5 (0.3%)	703 (47.4%)	429 (28.9%)	274 (18.5%)	774 (52.2%)	190 (12.8%)	54 (3.6%)	738 (49.8%)
Cereals & Other Grains	2106	23 (1.1%)	1175 (55.8%)	997 (47.3%)	178 (8.5%)	908 (43.1%)	675 (32.1%)	98 (4.7%)	583 (27.7%)
Dairy Products & Substitutes	2663	16 (0.6%)	634 (23.8%)	466 (17.5%)	168 (6.3%)	2013 (75.6%)	1196 (44.9%)	402 (15.1%)	892 (33.5%)
Desserts	924	4 (0.4%)	29 (3.1%)	9 (1%)	20 (2.2%)	891 (96.4%)	194 (21%)	460 (49.8%)	875 (94.7%)
Dessert Toppings & Fillings	121	0	9 (7.4%)	5 (4.1%)	4 (3.3%)	112 (92.6%)	9 (7.4%)	23 (19%)	112 (92.6%)
Eggs & Substitutes	80	0	52 (65%)	51 (63.8%)	1 (1.3%)	28 (35%)	21 (26.3%)	10 (12.5%)	0
Fats & Oils	1042	12 (1.2%)	334 (32.1%)	277 (26.6%)	57 (5.5%)	696 (66.8%)	647 (62.1%)	120 (11.5%)	117 (11.2%)
Fish, Seafood & Substitutes	581	2 (0.3%)	147 (25.3%)	121 (20.8%)	26 (4.5%)	432 (74.4%)	427 (73.5%)	57 (9.8%)	32 (5.5%)
Fruits & Fruit Juices	1519	3 (0.2%)	421 (27.7%)	363 (23.9%)	58 (3.8%)	1095 (72.1%)	10 (0.7%)	0	1091 (71.8%)
Legumes	205	0	132 (64.4%)	108 (52.7%)	24 (11.7%)	73 (35.6%)	69 (33.7%)	1 (0.5%)	3 (1.5%)
Meats & Substitutes	1436	4 (0.3%)	153 (10.7%)	134 (9.3%)	19 (1.3%)	1279 (89.1%)	1268 (88.3%)	293 (20.4%)	120 (8.4%)
Miscellaneous	783	4 (0.5%)	312 (39.8%)	171 (21.8%)	141 (18%)	467 (59.6%)	390 (49.8%)	43 (5.5%)	166 (21.2%)
Combination Dishes	1492	2 (0.1%)	39 (2.6%)	27 (1.8%)	12 (0.8%)	1451 (97.3%)	1445 (96.8%)	950 (63.7%)	783 (52.5%)
Nuts & Seeds	364	0	264 (72.5%)	206 (56.6%)	58 (15.9%)	100 (27.5%)	73 (20.1%)	20 (5.5%)	30 (8.2%)

TRA Category[1]	Total n	NA[2], n[3] (%)	Permitted[3] n (%)	Permitted[3] Exempt, n (%)	Permitted[3] Below M2K NPM[4], n (%)	Restricted[3] n (%)	Restricted[3] Exceed SOD, n (%)[5]	Restricted[3] Exceed SATFAT, n (%)[5]	Restricted[3] Exceed SUG, n (%)[5]
Potatoes	207	0	81 (39.1%)	36 (17.4%)	45 (21.7%)	126 (60.9%)	120 (58%)	16 (7.7%)	12 (5.8%)
Salads	141	0	7 (5%)	6 (4.3%)	1 (0.7%)	134 (95%)	116 (82.3%)	66 (46.8%)	57 (40.4%)
Sauces & Dips	1477	8 (0.5%)	81 (5.5%)	53 (3.6%)	28 (1.9%)	1388 (94%)	1316 (89.1%)	195 (13.2%)	588 (39.8%)
Snacks	900	3 (0.3%)	93 (10.3%)	24 (2.7%)	69 (7.7%)	804 (89.3%)	648 (72%)	327 (36.3%)	137 (15.2%)
Soups	702	0	27 (3.8%)	1 (0.1%)	26 (3.7%)	675 (96.2%)	665 (94.7%)	271 (38.6%)	210 (29.9%)
Sugars & Sweets	1579	2 (0.1%)	48 (3%)	13 (0.8%)	35 (2.2%)	1529 (96.8%)	59 (3.7%)	648 (41%)	1512 (95.8%)
Vegetables	1079	3 (0.3%)	539 (50%)	398 (36.9%)	141 (13.1%)	537 (49.8%)	512 (47.5%)	13 (1.2%)	135 (12.5%)
Foods for <4 years old	433	10 (2.3%)	197 (45.5%)	139 (32.1%)	58 (13.4%)	226 (52.2%)	32 (7.4%)	2 (0.5%)	211 (48.7%)
**Overall**	**24949**	**117 (0.5%)**	**5630 (22.6%)**	**4081 (16.4%)**	**1549 (6.2%)**	**19202 (77%)**	**12599 (50.5%)**	**5456 (21.9%)**	**10577 (42.4%)**

[1]Grocery food and beverage items in FLIP 2020 were categorized into food categories as defined in Health Canada's Table of Reference Amounts for Foods (TRA) [17]; [2]Foods and beverages that had insufficient nutrition information to determine if they would be permitted for or restricted from M2K were classified as NA. For example, if a product did not meet the exemption criteria, had nutrition information for serving size, sodium, and sugars available and did not exceed thresholds for either nutrient, but was missing information for saturated fat, the product could not be positively categorized as permitted for or restricted from M2K; thus, was classified as NA; [3]Percentage of total foods and beverages analyzed in that database; [4]Items that were below all three nutrient thresholds listed in Health Canada's M2K NPM were classified as permitted for M2K; [5]For the three nutrients, totals exceed 100% as some items exceed M2K NPM for more than one nutrient; Abbreviations: FLIP, Food Label Information and Price database; M2K, unhealthy foods/beverage Marketing-to-Kids; M2K NPM, Health Canada's proposed nutrient profile model for advertising restrictions; SOD, sodium threshold; SATFAT, saturated fat threshold; SUG, sugars threshold.

Supplementary Table S2. Summary of the proportion of chain restaurant menu items in the 2020 Canadian food supply that would exceed each nutrient threshold listed in Health Canada's M2K NPM [8] across menu categories.

Menu-FLIP 2020 Menu Categories[1]	n	NA[2], n[3] (%)	Permitted for Marketing to Children			Restricted from Marketing to Children			
			n (%)	Exempt, n (%)	Below M2K NPM[4], n (%)	n (%)	Exceed SOD, n (%)[5]	Exceed SATFAT, n (%)[5]	Exceed SUG, n (%)[5]
**Beverages**	**3307**	**0**	**587 (17.8%)**	**483 (14.6%)**	**104 (3.7%)**	**2720 (82.3%)**	**1127 (41.4%)**	**1130 (41.5%)**	**2682 (98.6%)**
Alcohol	68	0	25 (36.8%)	0	25 (36.8%)	43 (63.2%)	39 (90.7%)	4 (9.3%)	42 (97.7%)
Classic coffee/Teas	359	0	281 (78.3%)	259 (72.1%)	22 (22%)	78 (21.7%)	32 (41%)	49 (62.8%)	65 (83.3%)
Flavoured coffee/Teas	879	0	42 (4.8%)	0	42 (4.8%)	837 (95.2%)	469 (56.%)	633 (75.6%)	824 (98.5%)
Flavoured milk	170	0	0	0	0	170 (100%)	138 (81.2%)	147 (86.5%)	170 (100%)
Juices/Smoothies	772	0	5 (0.6%)	0	5 (0.7%)	767 (99.4%)	154 (20.1%)	82 (10.7%)	759 (99%)
Kids	57	0	8 (14%)	7 (12.3%)	1 (2%)	49 (86%)	26 (53.1%)	14 (28.6%)	47 (95.9%)
Milkshakes/Floats	207	0	0	0	0	207 (100%)	190 (91.8%)	201 (97.1%)	207 (100%)
Plain milk	39	0	38 (97.4%)	38 (97.4%)	0	1 (2.6%)	1 (100%)	0	1 (100%)
Soft drinks	715	0	147 (20.6%)	138 (19.3%)	9 (1.6%)	568 (79.4%)	78 (13.7%)	0	567 (99.8%)
Water	41	0	41 (100%)	41 (100%)	0	0	0	0	0
**Desserts**	**1722**	**2 (0.001%)**	**22 (1.3%)**	**0**	**22 (1.3%)**	**1698 (98.6%)**	**1102 (64.9%)**	**1110 (65.4%)**	**1611 (94.9%)**
Cookies	126	0	0	0	0	126 (100%)	82 (65.1%)	117 (92.9%)	122 (96.8%)
Donuts	137	0	0	0	0	137 (100%)	131 (95.6%)	125 (91.2%)	135 (98.5%)
Frozen desserts	597	0	19 (3.2%)	0	19 (3.2%)	578 (96.8%)	235 (40.7%)	232 (40.1%)	535 (92.6%)
Kids	105	2 (0.02%)	0.00%	0	0	103 (98.1%)	43 (41.8%)	56 (54.4%)	103 (100%)
Misc.	14	0	0.00%	0	0	14 (100%)	10 (71.4%)	12 (85.7%)	14 (100%)
Muffins	93	0	0.00%	0	0	93 (100%)	93 (100%)	42 (45.2%)	93 (100%)
Other baked goods	650	0	3 (0.5%)	0	3 (0.5%)	647 (99.5%)	508 (78.5%)	526 (81.3%)	609 (94.1%)
**Entrées**	**6827**	**2 (0.0003%)**	**82 (1.2%)**	**0**	**82 (1.2%)**	**6743 (98.8%)**	**6533 (96.9%)**	**5875 (87.1%)**	**649 (9.6%)**
Breakfast-Baked goods	133	0	0	0	0	133 (100%)	99 (74.4%)	109 (82%)	112 (84.2%)
Breakfast-Egg plates	236	0	0	0	0	236 (100%)	212 (89.8%)	236 (100%)	68 (28.8%)
Breakfast-Misc.	72	0	1 (1.4%)	0	1 (1.4%)	71 (98.6%)	52 (73.2%)	69 (97.2%)	19 (26.8%)
Breakfast-Sandwich/Wrap/Toast	186	0	0	0	0	186 (100%)	180 (96.8%)	177 (95.2%)	16 (8.6%)
Burgers	346	0	0	0	0	346 (100%)	338 (97.7%)	339 (98%)	32 (9.3%)
Hotdogs	29	0	0	0	0	29 (100%)	29 (100%)	29 (100%)	3 (10.3%)
Kids	264	1 (0.004%)	5 (1.9%)	0	5 (1.9%)	258 (97.7%)	244 (94.6%)	180 (69.8%)	56 (21.7%)
Meat only	122	0	0	0	0	122 (100%)	112 (91.8%)	116 (95.1%)	27 (22.1%)
Meat plate	163	0	0	0	0	163 (100%)	151 (92.6%)	153 (93.9%)	16 (9.8%)

			Permitted for Marketing to Children			Restricted from Marketing to Children			
**Menu-FLIP 2020 Menu Categories[1]**	**n**	**NA[2], n[3] (%)**	**n (%)**	**Exempt, n (%)**	**Below M2K NPM[4], n (%)**	**n (%)**	**Exceed SOD, n (%)[5]**	**Exceed SATFAT, n (%)[5]**	**Exceed SUG, n (%)[5]**
Misc.	15	0	0	0	0	15 (100%)	15 (100%)	15 (100%)	0
Other baked goods	81	0	0	0	0	81 (100%)	81 (100%)	80 (98.8%)	8 (9.9%)
Pasta	219	0	3 (1.4%)	0	3 (1.4%)	216 (98.6%)	211 (97.7%)	183 (84.7%)	8 (3.7%)
Pizza	2022	0	0	0	0	2022 (100%)	2018 (99.8%)	1904 (94.2%)	80 (4%)
Poultry only	260	0	1 (0.4%)	0	1 (0.4%)	259 (99.6%)	254 (98.1%)	202 (78%)	29 (11.2%)
Poultry plate	194	0	3 (1.5%)	0	3 (1.6%)	191 (98.5%)	177 (92.7%)	157 (82.2%)	13 (6.8%)
Poutine	286	0	0	0	0	286 (100%)	283 (99%)	286 (100%)	4 (1.4%)
Salad	286	1 (0.004%)	6 (2.1%)	0	6 (2.1%)	279 (97.6%)	261 (93.6%)	235 (84.2%)	42 (15.1%)
Sandwiches/Wraps	868	0	2 (0.2%)	0	2 (0.2%)	866 (99.8%)	862 (99.5%)	802 (92.6%)	75 (8.7%)
Seafood only	47	0	2 (4.3%)	0	2 (4.3%)	45 (95.7%)	41 (91.1%)	39 (86.7%)	2 (4.4%)
Seafood plate	384	0	37 (9.6%)	0	37 (9.6%)	347 (90.4%)	339 (97.7%)	164 (47.3%)	15 (4.3%)
Soup	183	0	0	0	0	183 (100%)	183 (100%)	72 (39.3%)	4 (2.2%)
Tacos/Burritos	273	0	4 (1.5%)	0	4 (1.5%)	269 (98.5%)	265 (98.5%)	245 (91.1%)	5 (1.9%)
Vegetarian	158	0	18 (11.4%)	0	18 (11.4%)	140 (88.6%)	126 (90%)	83 (59.3%)	15 (10.7%)
**Sides**	**2019**	**3 (0.002%)**	**1406.9%)**	**46 (2.3%)**	**94 (4.8%)**	**1876 (92.9%)**	**1797 (95.8%)**	**1014 (54.1%)**	**881 (47%)**
Bagels	88	0	0	0	0	88 (100%)	88 (100%)	38 (43.2%)	55 (62.5%)
Breads	247	0	8 (3.2%)	0	8 (3.2%)	239 (96.8%)	238 (99.6%)	86 (36%)	88 (36.8%)
Breakfast	198	1 (0.005%)	7 (3.5%)	0	7 (3.5%)	190 (96%)	169 (89%)	91 (47.9%)	130 (68.4%)
Cheese	54	0	3 (5.6%)	0	3 (5.6%)	51 (94.4%)	47 (92.2%)	50 (98%)	14 (27.5%)
Fries & Onion rings	321	2 (0.006%)	6 (1.9%)	0	6 (1.9%)	313 (97.5%)	300 (95.9%)	238 (76%)	141 (45.1%)
Fruit	22	0	15 (68.2%)	15 (68.2%)	0	7 (31.8%)	3 (42.9%)	1 (14.3%)	7 (100%)
Kids	86	0	12 (14%)	0	12 (14%)	74 (86.1%)	64 (86.5%)	20 (27%)	40 (54.1%)
Meat	81	0	0	0	0	81 (100%)	81 (100%)	55 (67.9%)	37 (45.7%)
Misc.	33	0	5 (15.2%)	0	5 (15.2%)	28 (84.9%)	26 (92.9%)	8 (28.6%)	11 (39.3%)
Other baked goods	91	0	1 (1.1%)	0	1 (1.1%)	90 (98.9%)	87 (96.7%)	59 (65.6%)	52 (57.8%)
Pasta	23	0	1 (4.3%)	0	1 (4.4%)	22 (95.7%)	22 (100%)	19 (86.4%)	13 (59.1%)
Potatoes(Non-Fried)	96	0	4 (4.2%)	0	4 (4.2%)	92 (95.8%)	90 (97.8%)	56 (60.9%)	31 (33.7%)
Poultry	138	0	0	0	0	138 (100%)	138 (100%)	95 (68.8%)	37 (26.8%)
Rice	47	0	17 (36.2%)	17 (36.2%)	0	30 (63.8%)	30 (100%)	11 (36.7%)	8 (26.7%)
Salad	216	0	27 (12.5%)	2 (0.9%)	25 (11.7%)	189 (87.5%)	177 (93.7%)	102 (54%)	103 (54.5%)
Seafood	49	0	13 (26.5%)	0	13 (26.5%)	36 (73.5%)	35 (97.2%)	10 (27.8%)	9 (25%)
Soup	97	0	0	0	0	97 (100%)	97 (100%)	32 (33%)	48 (49.5%)

Menu-FLIP 2020 Menu Categories[1]	n	NA[2], n[3] (%)	Permitted for Marketing to Children n (%)	Exempt, n (%)	Below M2K NPM[4], n (%)	Restricted from Marketing to Children n (%)	Exceed SOD, n (%)[5]	Exceed SATFAT, n (%)[5]	Exceed SUG, n (%)[5]
Vegetables	132	0	21 (15.9%)	12 (9.1%)	9 (7.5%)	111 (84.1%)	105 (94.6%)	43 (38.7%)	57 (51.4%)
**Starters**	**411**	**0**	**6 (1.5%)**	**1 (0.2%)**	**5 (1.2%)**	**405 (98.5%)**	**404 (99.8%)**	**311 (76.8%)**	**233 (57.5%)**
Breads	37	0	0	0	0	37 (100%)	37 (100%)	35 (94.6%)	16 (43.2%)
Cheese	17	0	0	0	0	17 (100%)	17 (100%)	17 (100%)	11 (64.7%)
Dips	37	0	0	0	0	37 (100%)	37 (100%)	34 (91.9%)	25 (67.6%)
Fries & Onion rings	42	0	0	0	0	42 (100%)	42 (100%)	35 (83.3%)	23 (54.8%)
Meat	24	0	0	0	0	24 (100%)	23 (95.8%)	19 (79.2%)	12 (50%)
Misc.	23	0	0	0	0	23 (100%)	23 (100%)	18 (78.3%)	16 (69.6%)
Poultry	69	0	0	0	0	69 (100%)	69 (100%)	62 (89.9%)	42 (60.9%)
Salad	15	0	3 (20%)	0	3 (20%)	12 (80%)	12 (100%)	6 (50%)	6 (50%)
Seafood	67	0	1 (1.5%)	0	1 (1.5%)	66 (98.5%)	66 (100%)	43 (65.2%)	47 (71.2%)
Soup	55	0	0	0	0	55 (100%)	55 (100%)	28 (50.9%)	17 (30.9%)
Vegetables	25	0	2 (8%)	1 (4%)	1 (4.2%)	23 (92%)	23 (100%)	14 (60.9%)	18 (78.3%)
**Overall**	**14286**	**7 (0.0005%)**	**837 (5.9%)**	**530 (3.7%)**	**307 (2.2%)**	**13442 (94.1%)**	**10963 (81.6%)**	**9440 (70.2%)**	**6056 (45.1%)**

[1]Menu items in Menu-FLIP 2020 were categorized into major and sub menu categories [16]; [2] Menu items that had insufficient nutrition information to determine if they would be permitted for or restricted from M2K were classified as NA. For example, if a menu item could *not* be reasonably assumed to have no added nutrients-of-concern, had nutrition information for serving size, sodium, and sugars available and did not exceed thresholds for either nutrient, but was missing information for saturated fat, the menu item could not be positively categorized as permitted for or restricted from M2K; thus, was classified as NA; [3]Percentage of total menu items analyzed in that database; [4]Items that were below all three nutrient thresholds listed in Health Canada's M2K NPM were classified as permitted for M2K; [5]For the three nutrients, totals exceed 100% as some items exceed M2K NPM for more than one nutrient; Abbreviations: Menu-FLIP, Food Label Information and Price chain restaurant menu database; M2K NPM, Health Canada's 2018 proposed nutrient profile model for advertising restrictions; SOD, sodium threshold; SATFAT, saturated fat threshold; SUG, sugars threshold.
